# Design and Optimization of Stacked Wideband On-Body Antenna with Parasitic Elements and Defected Ground Structure for Biomedical Applications Using SB-SADEA Method

**DOI:** 10.3390/bioengineering12020138

**Published:** 2025-01-31

**Authors:** Mariana Amador, Mobayode O. Akinsolu, Qiang Hua, João Cardoso, Daniel Albuquerque, Pedro Pinho

**Affiliations:** 1 Instituto de Telecomunicações, Departamento de Eletrónica, Telecomunicações e Informática, Universidade de Aveiro, 3810-193 Aveiro, Portugal; ptpinho@ua.pt; 2 Faculty of Arts, Computing and Engineering, Wrexham University, Wales LL11 2AW, UK; mobayode.akinsolu@glyndwr.ac.uk; 3 School of Computing and Engineering, University of Huddersfield, Huddersfield HD1 3DH, UK; q.hua@hud.ac.uk; 4 Instituto Superior de Engenharia de Lisboa, Departamento de Engenharia Eletrotécnica e de Computadores, 1959-007 Lisboa, Portugal; joaocardoso171@gmail.com; 5 Escola Superior de Tecnologia e Gestão de Águeda, Universidade de Aveiro, 3750-127 Águeda, Portugal; dfa@ua.pt

**Keywords:** on-body antenna, wideband antenna, antenna design, antenna optimization

## Abstract

The ability to measure vital signs using electromagnetic waves has been extensively investigated as a less intrusive method capable of assessing different biosignal sources while using a single device. On-body antennas, when directly coupled to the human body, offer a comfortable and effective alternative for daily monitoring. Nonetheless, on-body antennas are challenging to design primarily due to the high dielectric constant of body tissues. While the simulation process may often include a body model, a unique model cannot account for inter-individual variability, leading to discrepancies in measured antenna parameters. A potential solution is to increase the antenna’s bandwidth, guaranteeing the antenna’s impedance matching and robustness for all users. This work describes a new on-body microstrip antenna having a stacked structure with parasitic elements, designed and optimized using artificial intelligence (AI). By using an AI-driven design approach, a self-adaptive Bayesian neural network surrogate-model-assisted differential evolution for antenna optimization (SB-SADEA) method to be specific, and a stacked structure having parasitic elements and a defected ground structure with 27 tuneable design parameters, the simulated impedance bandwidth of the on-body antenna was successfully enhanced from 150 MHz to 1.3 GHz, while employing a single and simplified body model in the simulation process. Furthermore, the impact of inter-individual variability on the measured S-parameters was analyzed. The measured results relative to ten subjects revealed that for certain subjects, the SB-SADEA-optimized antenna’s bandwidth reached 1.6 GHz.

## 1. Introduction

The research community has been exploring the use of electromagnetic (EM) waves in emergent technologies developed to revolutionize biosignal sensing. EM-based systems enable simple, comfortable, and continuous vital sign monitoring, with a less complex and low-cost apparatus. In addition, non-invasive sensing allows one to keep track of the subject’s physiological condition with a realistic setup configuration, minimizing intrusiveness in their daily routine and leading to unbiased measures.

Popular methods for vital signs monitoring using electromagnetic (EM)-based systems include Doppler radar [1,2,3], ultrawideband radar [4], or WiFi (wireless fidelity) by analyzing channel state information [5]. Vital signs measurement is also feasible using RFID (radio frequency identification) tags [6] or on-body antennas, either using a single antenna [7] or a multi-input, multi-output (MIMO) approach [8,9]. Depending on the target application and the end-users, on-body sensors might be a more appropriate solution, and, in that case, it is important to reduce the number of sensors, simplifying the system hardware as much as possible.

The usage of a single on-body antenna to measure respiratory and cardiac signals was first introduced in [7]. The authors aimed to take advantage of application scenarios where the subject is already wearing on-body antennas for other purposes (e.g., a transmitter antenna of a communication system, such as a personal assistance system, or a portable radio) and verified if it was possible to extract vital signs with the available hardware. The working principle followed comprised sensing alterations in the antenna’s input impedance that occurred due to the thorax displacement over the antenna near-field. The biosignal waveforms were obtained by analyzing the phase of the S_11_ parameter. Three ultra-high-frequency (UHF) antennas were tested, specifically a meandered monopole operating at 370 MHz and two microstrip patch antennas operating at 900 MHz and 1.5 GHz, respectively. The authors concluded that it is possible to measure both respiratory and cardiac signals with this approach and the best results were achieved using the 370 MHz monopole antenna. Since the work was proposed as a proof of concept, the antennas were not specifically optimized for biosignal measurement and body motion artifacts occurring during the measurements were simply identified as a source of clutter, for which it is difficult to compensate.

Later, in [10], a study was conducted to determine the most suitable setup characteristics to measure vital signs with a single on-body antenna. The study involved simulations and practical measurements conducted on subjects to analyze the impact of operation frequency, antenna design, transmitted power, chest wall location, and distance between the antenna and the chest wall. Similar to [7], the author concluded that the accuracy decreased as the operation frequency increased, considering the cardiac signal in particular. The selected frequency was 2.45 GHz, since it is widely used in off-body communications and still provides enough sensitivity to measure vital signs with good accuracy. This selection was also supported by Hui et al. in [11], where the UHF band (0.3–3 GHz) was demonstrated to enable a strong energy coupling with minimal dispersion within the body. In [10], different antenna designs were tested (including monopoles, coplanar waveguides, loop, and microstrip patch antennas). Simulation results in [10] indicated that non-directive antennas were the most appropriate for biosignal measurements, showing a higher sensitivity to S_11_ phase variations. On the other hand, the practical measurements showed that non-directive antennas are more prone to body motion artifacts; therefore, microstrip patch antennas could be a better choice due to their better trade-off between accuracy and isolation. The importance of this trade-off is reported in [12], where a survey of on-body antenna arrays hig ighting future improvements emphasized the difficulty of matching antennas in contact with the human body and recommended wideband antennas that re-forced isolations using the ground plane.

Since the work conducted in [7,10] simply used an off-the-shelf approach, the antenna selection and associated operational frequencies were not optimized. However, in [10], it was observed that if the antenna is directly attached to the subject’s skin, an impedance mismatch occurs. Even though this mismatch can be addressed by keeping a certain distance between the antenna and the body, this solution is not always practical in a real setup, and the distance also has an impact on the assessment efficiency of the vital signs [10]. In this sense, one possible approach is matching the antenna with a human body model included in the simulation. In [13,14], two solutions were presented for on-body antennas matched considering a body model. A textile antenna was designed to operate at 433.92 MHz, validating the possibility of integrating these antennas directly with clothes [13], and a stacked microstrip antenna operating at 2.45 GHz was proposed in [14].

As opposed to the work carried out in [13], it should be noted that the impact of inter-individual variability in antenna impedance matching was addressed in [14] by aiming to simultaneously increase the antenna’s 10 dB impedance bandwidth, and maintain the antenna’s directivity to meet the requirements suggested in [10]. Through manual adjustment of different antenna parameters, a 10 dB impedance bandwidth of 170 MHz was achieved in [14]. This shows a significant increase in comparison to the 10 dB impedance bandwidth of 30 MHz achieved in [13]. Thus, the goal of this work, against the backdrop of the work in [14], is to fully explore the structure in [14] and propose a new stacked on-body microstrip antenna with parasitic elements and a defected ground structure that provides a wider 10 dB impedance bandwidth, while keeping the stacked structure as simple as possible for ease of integration in typical biomedical applications. For this purpose, a self-adaptive Bayesian neural network surrogate-model-assisted differential evolution for antenna optimization (SB-SADEA) method was employed to reach a 10 dB impedance bandwidth better than 1000 MHz, a more than 760% increase compared to the reference design in [14].

The SB-SADEA method is an artificial intelligence (AI)-based technique for efficient machine learning (ML)-assisted global optimization of contemporary antenna structures [15]. It belongs to the SADEA family of algorithms, which offer up to 20-times optimization speed improvements and yield design solutions of higher quality in comparison to conventional methods for antenna design and popular global optimization methods [16,17]. SB-SADEA does not rely on any ad hoc processes and initial design, making it more robust and suitable for a wider range of antenna design problems.

The outcomes from the SB-SADEA-driven optimization of the proposed stacked on-body microstrip antenna with parasitic elements and a defected ground structure indicate that such an approach can allow for the efficient adaptation of on-body antennas for different body parts using a general and simplified body model in simulations. Considering the use case in this work, the SB-SADEA-designed stacked on-body microstrip antenna with parasitic elements has been prototyped and its impedance matching tested using 10 subjects having body mass indexes (BMIs) varying between 18.7 kg/m^2^ and 24.7 kg/m^2^. This latter validation stage (after prototyping) was aimed at verifying the antenna’s robustness with respect to a wide population with different physical characteristics.

In this work, we introduce a novel AI-driven approach for the design and optimization of a stacked wideband on-body antenna tailored for biomedical applications.

The adoption of an AI-driven design approach in our work primarily aims to reduce time and costs, both in terms of computational resources and human effort, during the design and development of the proposed on-body antenna. While traditional antenna structures can often be optimized manually using design heuristics and simple parameter tuning to achieve satisfactory performance in terms of impedance bandwidth and radiation properties [18,19], this approach becomes increasingly laborious and impractical as the complexity of antenna structures grows [20,21,22]. Specifically, contemporary antennas are characterized by a high number of design parameters, which exert intricate and interdependent influences on the antenna’s frequency responses [23]. These implicit relationships make it extremely challenging, if not impossible, to achieve near-optimal design solutions using manual tuning or basic optimization techniques [24]. To address such challenges, design exploration through efficient optimization becomes necessary. However, as the number of design parameters, geometric constraints, and optimization goals increases, traditional optimization methods tend to fail or require prohibitively long computational times to achieve satisfactory results [19]. Furthermore, solving antenna optimization problems in high-dimensional design spaces presents significant challenges, even for existing optimization approaches incorporating AI techniques [15,17]. This necessitates the use of more efficient AI-driven optimization methods capable of addressing the complexities posed by modern antennas with high-dimensional design spaces. The SB-SADEA method, specifically developed for optimizing antenna structures with very high dimensionality, has demonstrated superior optimization capacity and efficiency compared to traditional and closely related methods [15]. Notably, the SB-SADEA method has been successfully validated in recent studies, where it efficiently optimized antenna structures involving over 100 design parameters and 70 specifications [21]. In the current work, the optimization of the on-body antenna involves 27 design parameters and 17 geometric constraints, necessitating an efficient and robust optimization approach to ensure geometric feasibility and high-performance design outcomes. Given its proven capabilities, SB-SADEA emerges as a natural choice for addressing this specific optimization problem.

Through the used SB-SADEA method, the antenna’s impedance bandwidth was significantly enhanced from 150 MHz to 1.3 GHz, with practical measurements reaching up to 1.6 GHz. The proposed design incorporates parasitic elements and a defected ground structure, ensuring robust performance across varying human body models and BMI ranges. The results hig ight the antenna’s potential for real-world applications, offering superior bandwidth, compactness, and adaptability compared to existing on-body antennas.

The remainder of this work is organized as follows: in Section 2, the antenna structure proposed in [14] is briefly described (from now on referred to as the *Reference design of the stacked antenna structure*) and the improvement goals are established; the working principle and the implementation of the SB-SADEA are presented in Section 3; Section 4 describes the configuration of the measurement setup and the sample population under study; Section 5 presents a discussion of the results; and concluding remarks are provided in Section 6.

## 2. Description of the Reference Design of the Stacked Antenna Structure

**Figure 1:**
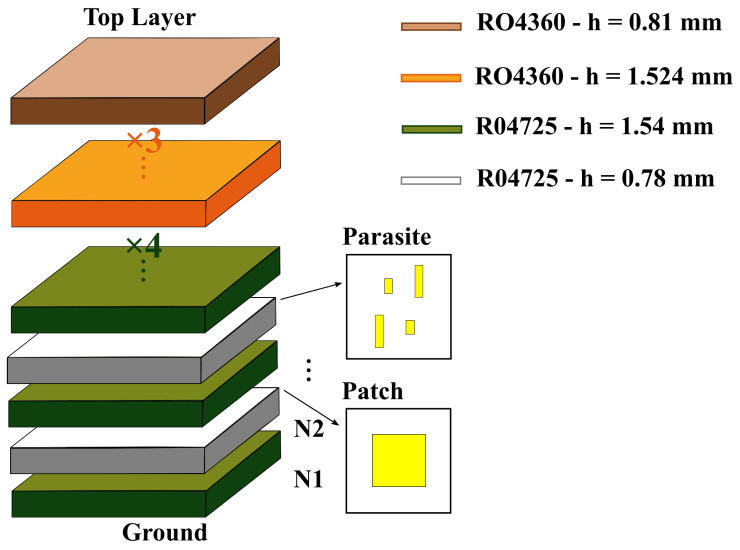
Schematics of the reference design of the stacked antenna structure layers [14].

The reference design of the stacked antenna structure was developed in [14] and it is composed of a multilayer structure as depicted in Figure 1. The antenna structure was inspired by [25]. The selection of a microstrip patch antenna for vital sign acquisition is primarily attributed to its proven accuracy in detecting physiological parameters, as hig ighted in [10]. These antennas are particularly suitable for such applications due to their ability to be optimized for increased bandwidth, ensuring proper matching under varying conditions. Additionally, they offer significant advantages for biomedical use, such as compact size, ease of fabrication, and seamless integration with wearable devices. This adaptability makes them a good choice for reliable vital sign monitoring across diverse user profiles. The main microstrip patch dictates the central frequency and the parasitic patches create a second resonance close to the central one, hence increasing the antenna’s impedance bandwidth [25]. Using the ground plane and the top layer orientation, it comprises a layer containing a regular microstrip patch operating at 2.45 GHz, a layer with four parasitic patches, a thick layer of dielectric superstrate, and a defected ground structure having two etched slots.

The antenna and parasitic patches lie on thick substrates, each composed of two layers of Rogers RO4725 with a relative permittivity ($\epsilon_{r}$) of 2.55 and a loss tangent (tanδ) of 0.0026 at 10 GHz. These substrates are depicted in green Figure 1, where green layers have a thickness (*h*) of 1.54 mm, and white layers have a thickness (*h*) of 0.78 mm. The superstrate block, located above the parasitic patches, is composed of four layers of Rogers RO4725 with a thickness (*h*) of 1.54 mm and four layers of Rogers RO4360 with $\epsilon_{r}=6.15$ and tan(δ)=0.0038 at 10 GHz, three of which have a thickness (*h*) of 1.524 mm (orange layers), and the last one has a thickness (*h*) of 0.81 mm (brown layer). These superstrate layers maintain the antenna’s impedance matching when close to the human body by smoot y decreasing the impact of the transition between different propagation media.

The use of slots in the ground plane is recommended in [26,27] to increase the antenna stability, since it reduces the $S_{11}$ parameter variation due to external factors. Additionally, these slots also increase the bandwidth without compromising the size of the antenna.

**Figure 2:**
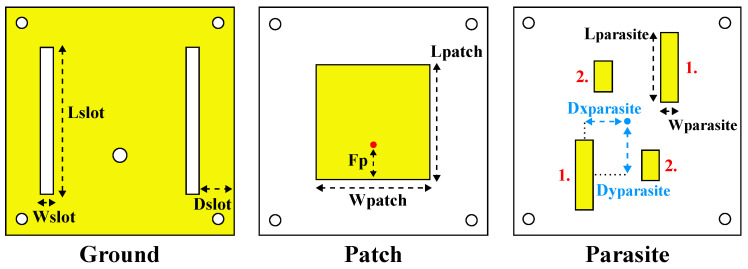
Design and parameters of the reference design of the stacked antenna structure [14]: the ground plane on the (**left**), the main patch at the (**center**), and the parasitic patches on the (**right**).

The designs corresponding to the ground plane, the main microstrip patch, and the parasitic patches are shown in Figure 2.

The optimization process followed in [14] consisted of a parameterization of 14 variables relative to the designs in Figure 2, plus the number of dielectric layers and their adjustments. The ground plane (Figure 2 (Ground)) has two rectangular slots to smoothen the magnitude of the in-band S_11_. The parameterization of the ground plane includes the length and width of the slots, as well as their horizontal position relative to the antenna margins. To reduce the optimization complexity, the parasitic patches (Figure 2 (Parasite)) were optimized in diagonal pairs, by adjusting their lengths, widths, and vertical and horizontal distances relative to the antenna’s center. Simulations were conducted with the antenna placed in contact with a human body model. The model had a cubic shape with a size of 304.58 mm × 221.08 mm × 336.83 mm, and its content included skin, fat, muscle, rib cage, visceral fat, lungs, and heart, all having dielectric properties at 2.45 GHz [28].

The physical dimensions of the reference design of the stacked antenna structure are $0.5151\lambda_{01}\times 0.5151\lambda_{01}$ (65 × 65 mm) with a thickness of 16.55 mm, considering that $\lambda_{01}$ is the vacuum wavelength at the least operational frequency in simulation (2.378 GHz). The final values of the design variables are reported in Table 1, where *Parasite 1* represents the diagonal with the largest parasitic patches and *Parasite 2* represents the diagonal with the smallest parasitic patches.

After a manual adjustment of the parameters, mostly via the sweeping of the parameters, the reference design of the stacked antenna structure showed an impedance bandwidth of 150 MHz. This impedance bandwidth, the bulkiness, and the weight of the reference design of the stacked antenna structure made it not an excellent candidate for the intended body-centric application. As a result, SB-SADEA was employed to take advantage of the possibility of tuning all the critical design variables over a much broader design space, allowing a fuller exploration of the potential of the topological evolution of the antenna structure. The SB-SADEA-driven design exploration aims to meet three major objectives: (1) increase the antenna’s impedance bandwidth (to have at least a 10 dB impedance bandwidth of 1 GHz, covering the UHF spectrum of 2 GHz to 3 GHz); (2) simplify the antenna design to allow for ease of prototyping and integration with other systems for biomedical applications; and (3) reduce the bulkiness or lower the profile of the antenna. The SB-SADEA-driven optimization of the antenna is described in the next section (i.e., Section 3).

## 3. SB-SADEA-Driven Optimization

**Figure 3:**
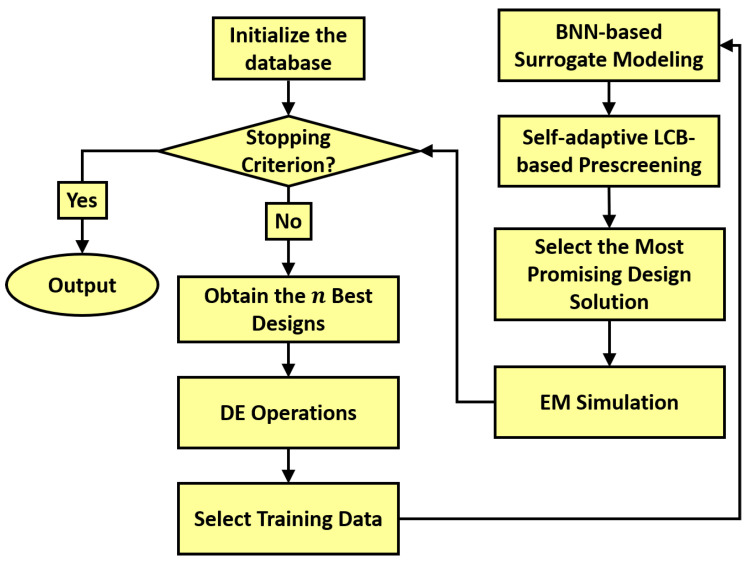
SB-SADEA flow diagram [15].

The SB-SADEA method works as shown in Figure 3. It is initialized by sampling the design space of the on-body antenna using the Latin hypercube sampling method [29]. Full-wave EM simulations are then carried out on the samples to create the initial database, comprising the candidate designs and their simulation results. If the preset stopping criterion such as the maximum number of EM simulations is met, then the best candidate design solution is outputted from the database; otherwise, SB-SADEA iterates over the following sequential steps: (1) Formulation of the population using the *n* best designs from the database. (2) Application of the differential evolution (DE) operations to the formulated population to generate new child solutions. (3) Obtaining the nearest (measured as the Euclidean distance) samples for each child solution as the training data points and construction of a Bayesian neural network (BNN)-based surrogate model. (4) Self-adaptive lower confidence bound (LCB)-based prescreening of the generated child solutions. (5) Simulation of the estimated best child solution and adding it and its performance values to the database. In comparison to other methods in the SADEA family [16,17,30,31,32], it should be noted that the efficiency improvement of SB-SADEA mainly stems from the use of the BNN for surrogate modeling and self-adaptive LCB for the prescreening of predictions [15]. More details about the SB-SADEA method can be found in [15].

For the SB-SADEA-driven optimization of the on-body antenna, a population size of 100 was used, and all other algorithmic settings were the default settings in [15]. The individual positions and sizes of each parasitic patch were considered to be the most critical design parameters concerning the antenna’s impedance bandwidth. Therefore, a total of 27 variables (as shown in Table 2 and Figure 1 and Figure 2) were considered, where 22 of the design parameters are relative to the antenna’s elements (i.e., the ground plane, main patch, and parasitic patches) and the remaining five parameters are relative to the number of the dielectric layers of the substrate and superstrate (i.e., variables N1, N3, N5, N6, and N7, corresponding to the layer number, starting from the ground layer and moving upward). The search ranges of the design parameters are shown in Table 2. To ensure geometric congruity in all possible cases during the optimization process, the following geometric constraints are used:

(1) Dslot+Wslot<(1.01×Wpatch)−6.3mm

(2) Dxparasite1<Wpatch

(3) Dxparasite2<Wpatch

(4) Dxparasite3<Wpatch

(5) Dxparasite4<Wpatch

(6) Dyparasite1<Lpatch

(7) Dyparasite2<Lpatch

(8) Dyparasite3<Lpatch

(9) Dyparasite4<Lpatch

(10) $$\begin{array}{c} Dxparasite1+Wparasite1<\ldots \\ (1.01\times Wpatch)-6.3\;\mathrm{mm} \end{array}$$

(11) $$\begin{array}{c} Dxparasite2+Wparasite2<\ldots \\ (1.01\times Wpatch)-6.3\;\mathrm{mm} \end{array}$$

(12) $$\begin{array}{c} Dxparasite3+Wparasite3<\ldots \\ (1.01\times Wpatch)-6.3\;\mathrm{mm} \end{array}$$

(13) $$\begin{array}{c} Dxparasite4+Wparasite4<\ldots \\ (1.01\times Wpatch)-6.3\;\mathrm{mm} \end{array}$$

(14) $$\begin{array}{c} Dyparasite1+Lparasite1<\ldots \\ (1.01\times Wpatch)-6.3\;\mathrm{mm} \end{array}$$

(15) $$\begin{array}{c} Dyparasite2+Lparasite2<\ldots \\ (1.01\times Wpatch)-6.3\;\mathrm{mm} \end{array}$$

(16) $$\begin{array}{c} Dyparasite3+Lparasite3<\ldots \\ (1.01\times Wpatch)-6.3\;\mathrm{mm} \end{array}$$

(17) $$\begin{array}{c} Dyparasite4+Lparasite4<\ldots \\ (1.01\times Wpatch)-6.3\;\mathrm{mm} \end{array}$$

The optimization goal is to find the largest possible 10 dB impedance bandwidth in the UHF spectrum with 2 GHz to 3 GHz being the primary frequency range of interest. To better manage the potential degradation of the antenna’s impedance bandwidth profile after physical implementation, a 12 dB target was set as the maximum allowable in-band return loss for the SB-SADEA-driven optimization.

To reduce the cost of each EM simulation, the human body model was replaced by a simpler version. The simplified version of the human body model comprised three layers with a size of 350 mm × 350 mm, where the skin layer, the fat layer, and the muscle had thicknesses (*h*) of 2.3 mm, 7 mm, and 15 mm, respectively. Using a mesh density of 15 cells per wavelength to have about 7,854,000 hexahedral mesh cells for the discretization of the overall antenna model in CST Microwave Studio Suite, each simulation costed about 8 min (from a wall clock) on average on a workstation having an Intel eight-core i9-9900K 3.6 GHz CPU, and 32 GB RAM CPU. The solver used was the time domain solver with a finite integration technique implementation and an accuracy of −40 dB.

**Figure 4:**
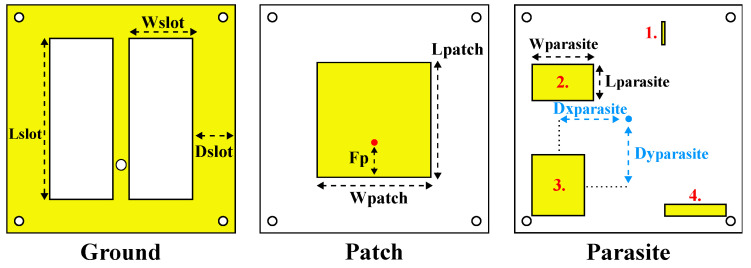
Schematics of antenna structure after optimization process.

**Figure 5:**
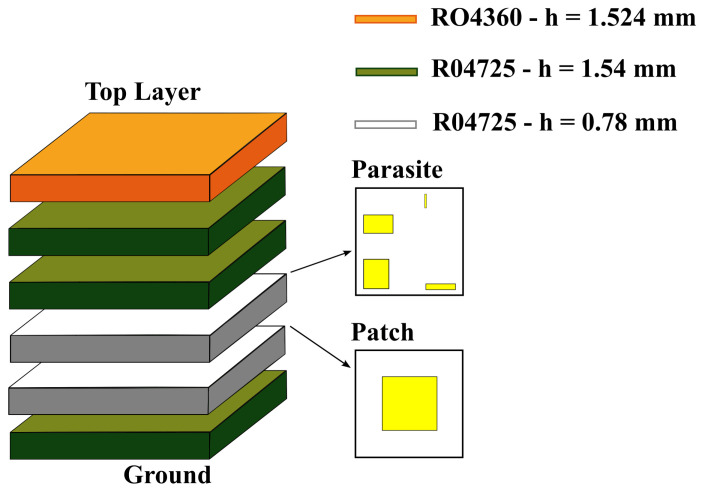
Ground plane and parasitic patches design after optimization process.

After 512 EM simulations costing about 3 days, SB-SADEA obtained a design covering the impedance bandwidth of interest in the UHF spectrum. The optimized values for the SB-SADEA-optimized design are reported in Table 2 and its physical implementation and its measurement results are discussed in Section 4. The physical dimensions of the SB-SADEA-optimized version of the antenna are $0.5093\lambda_{02}\times 0.5093\lambda_{02}$ (86.53 × 86.53 mm) with a thickness of 7.74 mm, considering that $\lambda_{02}$ is the vacuum wavelength at the least operational frequency in simulation (1.766 GHz). The final design and structure are shown in Figure 4 and Figure 5, respectively.

**Figure 6:**
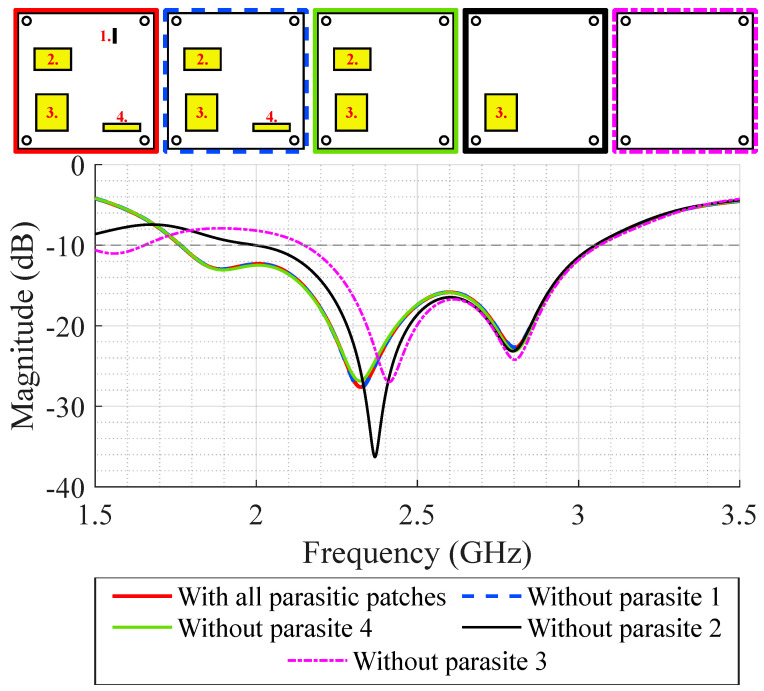
Individual contribution of the parasitic patches on the simulated S_11_.

A comparative analysis of the SB-SADEA method was conducted against other traditional antenna optimization techniques commonly implemented in commercial CAD/CEM tools and widely utilized by antenna designers. Specifically, the SB-SADEA method was compared with two prominent built-in optimization methods available in the CST Microwave Studio Suite: particle swarm optimization (PSO) and the trust region framework (TRF). The choice of these methods was guided by their established popularity both in the literature and among practicing antenna designers [19,22,33]. Using the same optimization goal and computational budget (i.e., 1500 EM simulations) as SB-SADEA, both PSO and TRF were unable to achieve a design solution that fully satisfied the target impedance bandwidth. Specifically, PSO and TRF exhibited violations of approximately 0.04 dB and 0.89 dB, respectively, with PSO requiring 1500 EM simulations and TRF obtaining its best result using 644 EM simulations. Given that the violation observed in the PSO result (0.04 dB) was relatively minor and close to the target (0 dB), it was considered a successful outcome and was directly compared with the successful result of SB-SADEA. This comparison hig ights that SB-SADEA achieves a threefold improvement in optimization speed. These findings align with our observations in the literature [19], which motivated the adoption of the AI-driven SB-SADEA method over conventional optimization approaches to significantly reduce design time while ensuring higher-quality design solutions. Characterization of the SB-SADEA-optimized antenna design was carried out to understand the contribution of each parasitic patch to the antenna’s 10 dB impedance bandwidth. For this purpose, the parasitic patches were removed from the design one by one, and the S_11_ parameter was evaluated for each case. During the optimization process, multiple configurations of parasitic elements were investigated to assess their influence on the antenna’s performance. These included variations in the number, placement, and orientation of parasitic stubs. Among these, the configuration shown in Figure 6 shows the most significant improvement in terms of bandwidth and impedance matching, making it the most suitable candidate for the final design. While other variants showed marginal improvements or no notable benefit, their detailed results were available upon request for further reference.

From Figure 6, it can be seen that if all the parasitic patches are removed from the design, the antenna’s 10 dB impedance bandwidth is 914 MHz. This effect can be attributed to the increase in the dimensions of ground plane slots, from 41.8 mm × 3.5 mm to 61.13 mm × 25.34 mm. So, one can conclude that the parasitic patches have an important impact on the antenna’s 10 dB impedance bandwidth. Specifically, the presence of parasitic patches number 2 and 3 further increased the antenna’s 10 dB impedance bandwidth by approximately 400 MHz according to Figure 6. On the other hand, parasitic patches number 1 and 4 have a minimal impact; thus, the design can be simplified by removing these latter ones.

**Figure 7:**
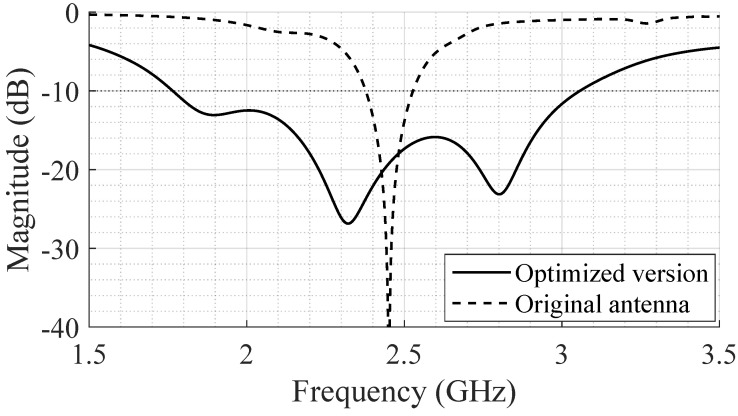
Comparison of the simulated S_11_ between the reference design of the stacked antenna structure (identified in the graph as the *Original Antenna*) and the SB-SADEA-optimized antenna version.

Figure 7 shows the comparison of the simulated S_11_ for the final SB-SADEA-optimized design (exclusively with parasitic patches number 2 and 3) and the reference design of the stacked antenna structure. In total, the bandwidth increased from 150 MHz to 1.3 GHz, reaching the desired goal.

**Figure 8:**
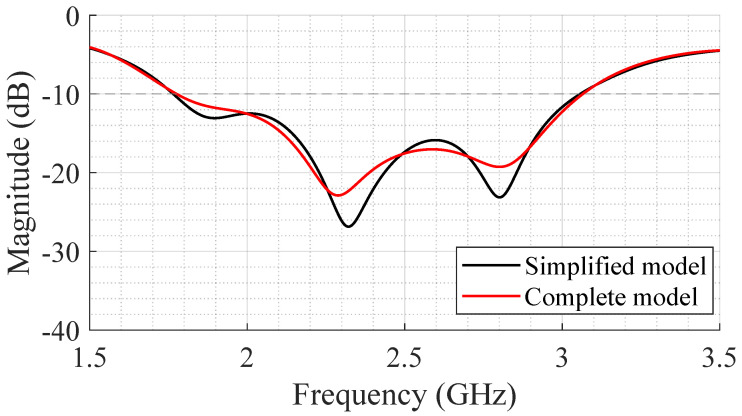
Impact of human body model used in simulations on S_11_ parameter.

Note that the antenna was optimized using a simplified human body model to reduce the simulation time and overall computational cost. So, to evaluate the impact of the model used in simulation, the SB-SADEA-optimized antenna was simulated using a more complete model, specifically the model used in [14]. Figure 8 shows a comparison of the S_11_ using a simplified model and a more complete model. One can observe that there are some distinctions, but they are not substantial. The 10 dB impedance bandwidth remains approximately the same and the magnitude of the S_11_ parameter increased by less than 5 dB.

## 4. Setup Description for Practical Measurements

**Figure 9:**
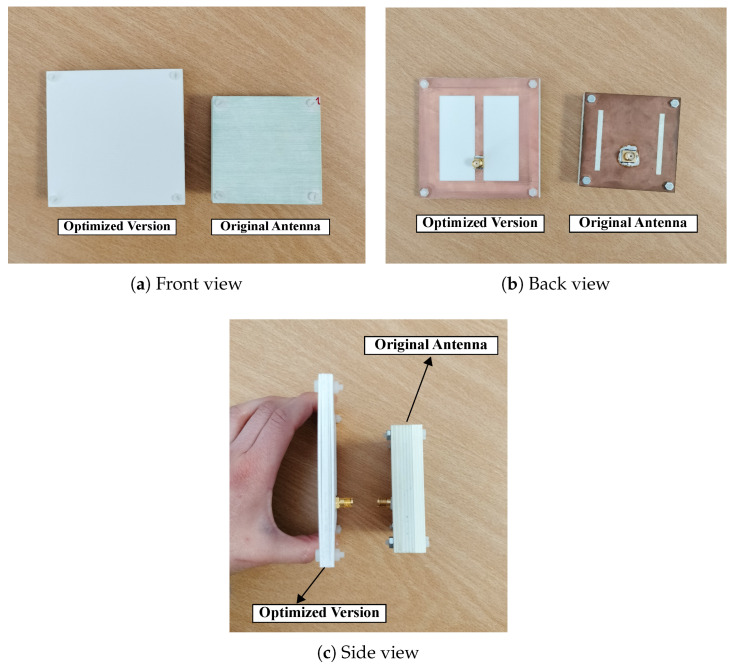
Manufactured antennas. The *Optimized Version* corresponds to the SB-SADEA-optimized antenna and the *Original Antenna* corresponds to reference design of stacked antenna structure.

After the SB-SADEA-driven optimization process and the characterization of the on-body antenna via simulations, the antenna was manufactured. Figure 9 shows the reference design of the stacked antenna structure and the SB-SADEA-optimized version. After prototyping, it can be seen that the thickness of the reference design of the stacked antenna structure decreased from 16.55 mm to 7.74 mm. Its remaining dimensions increased from 65 mm × 65 mm to 86.53 mm × 86.53 mm, which was expected since the least operational frequency of the SB-SADEA-optimized antenna (1.766 GHz) is lower than that of the reference design (2.378 GHz). Therefore, the dimensions in relation to each wavelength remained approximately the same (specifically $0.5151\lambda_{01}\times 0.5151\lambda_{01}$ for the reference design of the stacked antenna structure and $0.5093\lambda_{02}\times 0.5093\lambda_{02}$ for the SB-SADEA-optimized antenna).

The S_11_ parameter of the SB-SADEA-optimized antenna was measured using 10 different subjects to evaluate the robustness of the impedance bandwidth when considering different body structures. The same procedure was conducted for the reference design of the stacked antenna structure. Table 3 presents the characteristics of the subjects included in this study, namely, gender and body mass index (BMI).

**Figure 10:**
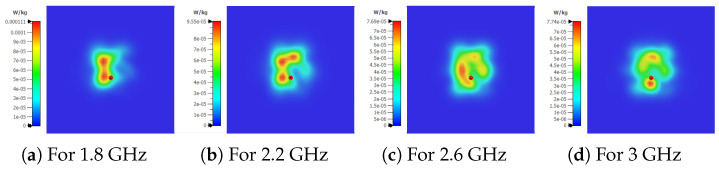
Simulated SAR for all bandwidth of SB-SADEA-optimized antenna version.

The S_11_ measurements were performed using the PNA-X N5242A from Keysight Technologies. An input power of -20 dBm was used because it is within the acceptable power range suggested by [10] and concurrently assures the subjects’ safety. This latter aspect was verified through the analysis of the specific absorption rate (SAR) over the antenna’s impedance bandwidth via simulations. Figure 10 shows that the highest absorption occurs at 1.8 GHz being 0.111 mW/kg and a lower absorption occurs at the center frequency (2.45 GHz), namely, 0.0767 mW/kg—this being approximately the same at 2.6 GHz and 3 GHz. According to the simulation results, an input power of −20 dBm assures the subjects’ safety since it leads to an absorption far below the limits suggested by the International Commission for Non-Ionizing Radiation Protection (ICNIRP) [34].

**Figure 11:**
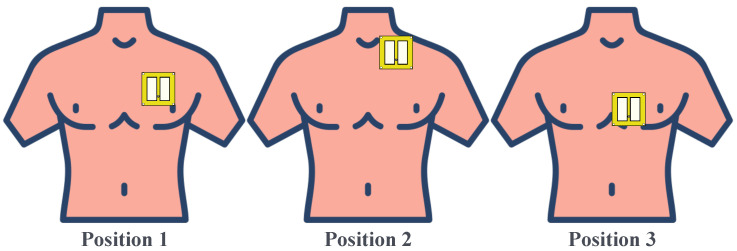
Schematics of the measurement setup for three different variants of the antenna position.

Practical measurements were carried out by measuring the antenna’s S_11_ parameter while placing the antenna on the subject’s chest wall in contact with the skin. In this setup, it was difficult to guarantee the same measurement conditions for all subjects since the antenna was not always placed exactly in the same position for all the subjects. This could compromise the reproducibility of the results and does not allow for a fair comparison of the results. Following the same approach as in [14], the proposed broadband antenna detects vital signs, such as respiratory and cardiac motion, by measuring variations in the phase of the S_11_ parameter. Respiratory movements alter the size and dielectric properties of the lungs, while cardiac motion induces changes in the dimensions of the heart and the movements of the chest wall. These variations affect the antenna’s near-field interaction with the chest layers, resulting in measurable phase shifts in the S_11_ parameter. Furthermore, the human body model used in the optimization process was a simplified version, which represents equally all body parts and does not generalize to all populations. For this reason, some discrepancies between simulations and practical results are expected. To account for the expected variability in the measurements, the S_11_ parameter was measured in three different positions along the chest wall, as depicted in Figure 11. This allowed for the characterization of the antenna performance considering intra-individual variability.

Is important to notice that the placement and attachment of on-body antennas are known to significantly influence the S_11_ parameter and the quality of signal acquisition, as observed in related studies [14]. In this work, the antenna was manually placed on the body, and slight variations in pressure were observed during the measurements. Future experiments will include standardized attachment mechanisms to ensure consistent contact pressure and placement, which are expected to improve measurement accuracy and repeatability.

## 5. Results and Discussion

**Figure 12:**
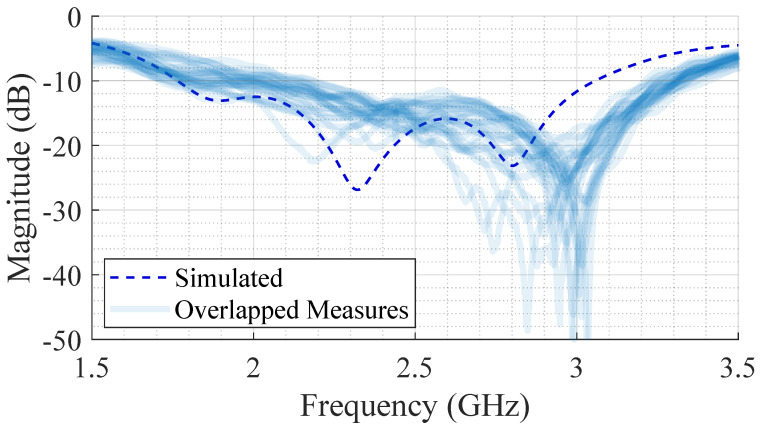
Global analysis of the measured $S_{11}$ for the SB-SADEA-optimized version of the antenna.

Figure 12 shows the measured S_11_ parameter for all subjects in all positions superimposed, in comparison with the simulated values. From Figure 12, it can be seen that there is a slight shift in the central frequency towards a higher value (namely, to approximately 2.7 GHz). This effect might be related to two possible causes: (1) the individual variability due to different body structures (discussed in detail afterwards); and (2) eventual irregularities in the antenna manufacturing such as fabrication tolerances. The antenna is a relatively complex structure, composed of stacked dielectric layers, and their arrangement might easily lead to uneven air gaps between the stacked layers. However, the shift is not significant and the antenna is still well matched at the central frequency and over the entire bandwidth of interest.

**Figure 13:**
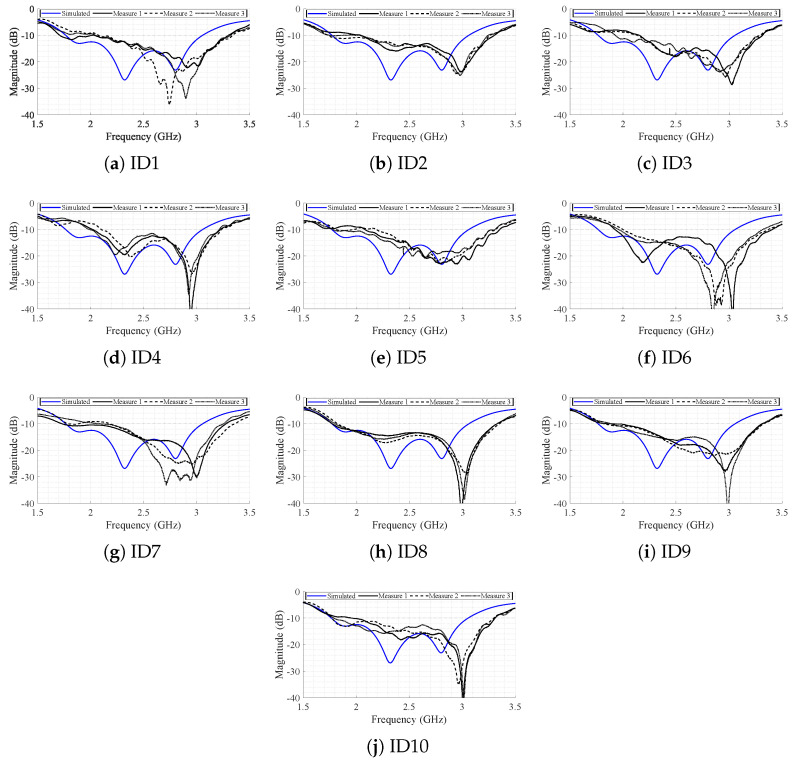
Individual analysis of the measured S_11_ for the SB-SADEA-optimized version of the antenna.

In Figure 12, it can also be seen that each S_11_ parameter trace presents a different bandwidth and these variations are more pronounced at the lower frequency bound. Figure 13 shows the measured S_11_ parameter for each subject, measured several times. By observing each subject in turn, it can be seen that different measurement positions provide different S_11_ parameter traces, as was expected since the human body is a complex and irregular structure that is quite difficult to represent with a single model in simulation. Subjects with low BMI present a higher variability for the resonance frequency values between the measured positions, as is the case of ID1 (BMI 18.7), ID6 (BMI 20.1), or ID7 (BMI 22.1). On the other hand, subjects with a higher BMI present approximately the same S_11_ parameter trend for different positions (ID2, ID4, and ID5).

**Figure 14:**
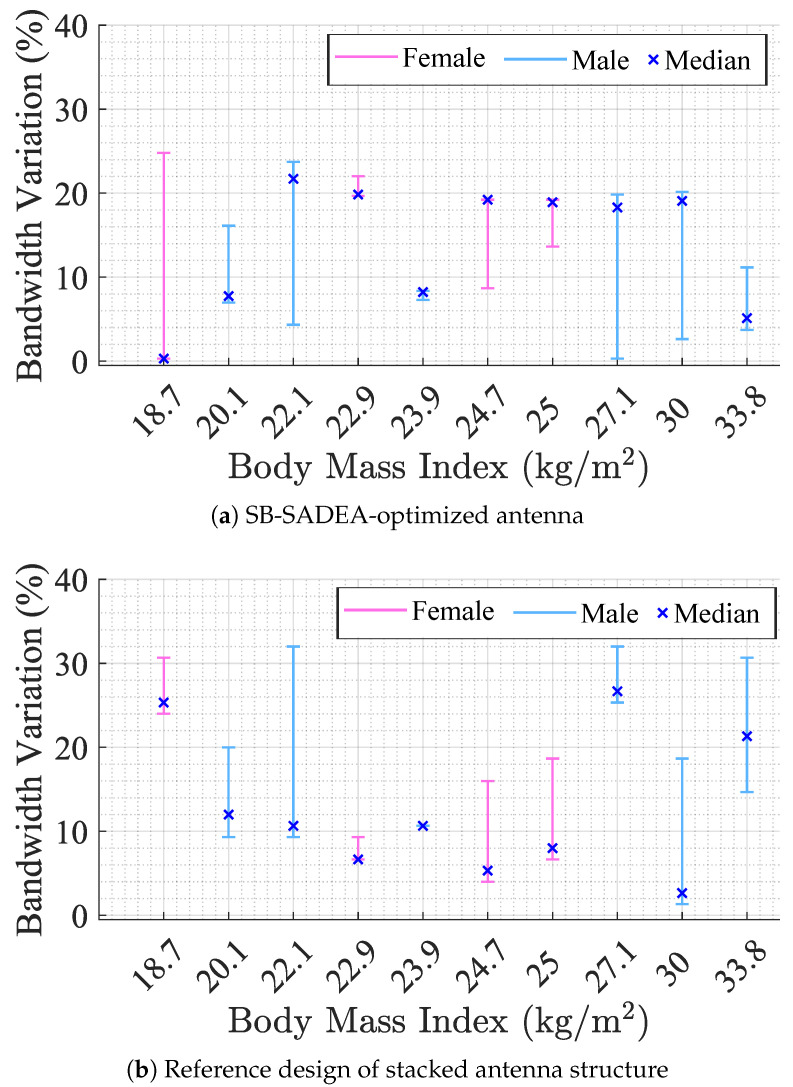
Percentage of bandwidth variation in relation to simulation for different measurement positions.

Figure 14a shows the percentage of 10 dB impedance bandwidth variation relative to the expected one (obtained in simulation, namely, 1.3 GHz), according to BMI and gender. The variation is expressed in absolute value, where the upper bound corresponds to +24.8% and the lower bound is −11.2%. The exact bandwidth values for each subject on each position are presented in Table 3. Subjects with extreme BMI present a bandwidth similar to the simulated one, namely, ID1 (in two positions), and ID2, ID4, and ID5 (all in one position). The remaining subjects present a mean bandwidth variation of 12.5%.

Most subjects present the same bandwidth in two positions and a different one in the remaining one, which justifies the high-variation ranges observed in Figure 14a. There is no relation between the range of variation and BMI, nor with gender. Nonetheless, it can be seen that subjects with average BMI (ID3 and ID8) are the ones with more consistency between the three measured positions. In general, the measured bandwidth results surpassed their simulated values, even reaching a maximum of 1.6 GHz and a median of 1.45 GHz in all positions.

The same study was conducted using the reference design of the stacked antenna structure on the same subjects. Figure 14b shows the percentage of bandwidth variation relative to the simulation results. In this case, a higher variation was observed relative to the simulated bandwidth, where the upper bound corresponds to +32% and the lower bound is −20%. Similar to the SB-SADEA-optimized antenna case, ID3 and ID8 are the ones with more consistency between the three measured positions. This time, only ID2 presents a 10 dB impedance bandwidth close to the simulated one, where the remaining values were on average 171.5 MHz. Finally, the maximum measured 10 dB impedance bandwidth did not surpass 198 MHz.

The optimized version of the antenna incorporates larger ground plane notches to achieve enhanced impedance bandwidth. However, this modification may influence back radiation, potentially affecting measurement accuracy and increasing energy absorption by human tissues. Although back radiation is less critical in applications focusing on near-field interaction, it can still play a role in ensuring user safety and mitigating interference from nearby objects. Future work will investigate this aspect in detail and explore potential design improvements. These may include adjustments to the ground plane to reduce back radiation.

## 6. Conclusions

In this work, the SB-SADEA method has been used to optimize a stacked on-body antenna with parasitic elements and a defected ground structure, aiming to take advantage of the antenna’s structure to maximize its impedance bandwidth for biomedical applications. Table 4 presents a comparision between the work developed in this article and other works showing the strengths of the antenna design optimized with the AI-driven SB-SADEA method, which sets it apart from other work in the field on on-body biomedical antennas. This design achieves a superior balance regarding compact size (50 × 40 × 1.6 mm), wideband performance (1.3–1.6 GHz), and robustness to variability, making it hig y suitable for real-world applications in vital sign monitoring, particularly for respiratory and cardiac motion detection. Compared to non-AI-optimized designs, the antenna demonstrates significant improvements in design efficiency. The implementation of the SB-SADEA-driven optimization of the proposed antenna provides insights into a new approach for the design of on-body antennas; it can also be applied to other body parts while using a simple human body model. Considering the intended on-body vital signs acquisition application, the main goal was achieved by increasing the antenna’s impedance bandwidth up to 1300 MHz in comparison to the reference antenna’s impedance bandwidth of 150 MHz. Then, the inter- and intra-individual variabilities were investigated and compared for the manufactured prototypes of both antennas by measuring the S_11_ using 10 subjects with different BMI values. It was observed that the body structure affects the antenna’s impedance bandwidth but generally provides a higher impedance bandwidth in comparison to the simulated results. The measurement results also showed that the reference antenna did not surpass an impedance bandwidth of 198 MHz, while the SB-SADEA-optimized antenna achieved an impedance bandwidth of 1600 MHz, depending on the subject and location of measurements. The SB-SADEA-optimized antenna’s impedance bandwidth remained fairly stable for different chest wall positions for subjects having average BMI values in the range of 22–24 kg/m^2^, while it varied for subjects having extreme BMI values in the range of 18–22 kg/m^2^ and 24–34 kg/m^2^.

In the future, additional work focused on lowering the profile of the antenna by reducing its dimensions while maintaining the impedance bandwidth will be carried out. Also, to achieve a real-world physical implementation and utilization of the on-body antenna, the use of alternative substrates such as textiles will be investigated to ascertain the viability of the on-body antenna for other wearable applications.

## Figures and Tables

**Table 1:** Reference design of the stacked antenna structure dimensions in [mm].

Dslot	Lslot	Wslot	Wpatch	Lpatch	FP	Lparasite1
19	41.8	3.5	32.48	32.77	6.6	20.05
Wparasite1	Dxparasite1	Dyparasite1	Lparasite2	Wparasite2	Dxparasite2	Dyparasite2
5.16	19.11	16.78	8.66	4.94	8.34	10.64

**Table 2:** Search space for the antenna design parameters and their optimal values obtained by SB-SADEA. All parameters are continuous variables with dimensions in mm, except N1, N3, N5, N6, and N7, which take values in {0, 1}, {0, 1}, {0, 1, 2, 3, 4}, {0, 1, 2, 3}, and {0, 1}, respectively.

Design Parameter and Search Range	SB-SADEA Optimum
Dslot [3.12 to (1.01 × Wpatch) − 6.3]	3.12
Lslot [0 to (2.01 × Wpatch) − 12.6]	61.13
Wslot [0 to (1.01 × Wpatch) − 9.42]	25.34
Wpatch [30.5 to 52.3]	43.05
Lpatch [30.6 to 45.3.3]	45.58
FP [3 to 23.15]	5.25
Lparasite1 [0.2 to (1.01 × Wpatch) − 6.3]	8.31
Wparasite1 [0.2 to(1.01 × Wpatch) − 6.3]	0.50
Dxparasite1 [Wparasite1 to (1.01 × Wpatch) − 6.3]	12.84
Dyparasite1 [Lparasite1 to (1.01 × Wpatch) − 6.3]	28.55
Lparasite2 [0.2 to (1.01 × Wpatch) − 6.3]	14.01
Wparasite2 [0.2 to (1.01 × Wpatch) − 6.3]	23.53
Dxparasite2 [Wparasite2 to (1.01 × Wpatch) − 6.3]	13.33
Dyparasite2 [Lparasite2 to (1.01 × Wpatch) − 6.3]	7.01
Lparasite3 [0.2 to (1.01 × Wpatch) − 6.3]	23.56
Wparasite3 [0.2 to (1.01 × Wpatch) − 6.3]	20.17
Dxparasite3 [Wparasite3 to (1.01 × Wpatch) − 6.3]	16.70
Dyparasite3 [Lparasite3 to (1.01 × Wpatch) − 6.3]	13.30
Lparasite4 [0.2 to (1.01 × Wpatch) − 6.3]	4.51
Wparasite4 [0.2 to (1.01 × Wpatch) − 6.3]	23.12
Dxparasite4 [Wparasite4 to (1.01 × Wpatch) − 6.3]	13.74
Dyparasite4 [Lparasite4 to (1.01 × Wpatch) − 6.3]	32.36
N1 {0,1}	1
N3 {0,1}	0
N5 {0,1,2,3,4}	2
N6 {0,1,2,3}	1
N7 {0,1}	0

**Table 3:** Description of subjects and impedance bandwidth variation of SB-SADEA-optimized antenna for different measurement positions.

Subject ID	Gender	BMI (kg/m^2^)	Bandwidth (GHz)		
			P1	P2	P3
ID1	F	18.7	1.610	1.286	1.294
ID2	M	30	1.324	1.550	1.536
ID3	M	23.9	1.184	1.196	1.398
ID4	M	33.8	1.224	1.146	1.242
ID5	M	27.1	1.286	1.526	1.546
ID6	M	20.1	1.498	1.390	1.380
ID7	M	22.1	1.570	1.596	1.234
ID8	F	22.9	1.574	1.544	1.546
ID9	F	25	1.538	1.534	1.466
ID10	F	24.7	1.402	1.538	1.538

M—Male; F—Female; BMI—Body Mass Index; P1—Position 1; P2—Position 2; P3—Position 3.

**Table 4:** Comparison of on-body antennas for biomedical applications.

Reference	Application	Optimization	Bandwidth (GHz)	Features	Advantages	Size
This Work	On-body biomedical antennas.	SB-SADEA	1.4 (1.7–3.1)	Wideband, optimized for biomedical applications.	Superior bandwidth, AI-driven for speed, robust to user variability.	50 × 40 × 1.6
[35]	Military healthcare	-	center frequency 2.4	Integrated into uniforms, robust in military environments.	Real-time health monitoring, robust to interference.	35.2 × 31.6 × 1.6
[13]	On-body cardiopulmonary monitoring	-	center frequency 0.433	Textile-based, comfortable, designed for respiratory monitoring.	Non-intrusive, low-frequency operation.	70 × 70 × 5.3
[36]	On-body ISM communications	Data-Driven Evolutionary Algorithm	1.8 (24–25.8)	Flexible substrate, bending resilience, high gain for ISM bands.	High gain and bandwidth for on-body ISM applications.	30 × 25 × 0.5

## Data Availability

The original contributions presented in this study are included in the article. Further inquiries can be directed to the corresponding author(s).
